# The Severity of *Osteogenesis Imperfecta* and Type I Collagen Pattern in Human Skin as Determined by Nonlinear Microscopy: Proof of Principle of a Diagnostic Method

**DOI:** 10.1371/journal.pone.0069186

**Published:** 2013-07-15

**Authors:** Javier Adur, Lilia DSouza-Li, Marcus Vinícius Pedroni, Carlos E. Steiner, Vitor B. Pelegati, Andre A. de Thomaz, Hernandes F. Carvalho, Carlos L. Cesar

**Affiliations:** 1 Biophotonic Group, Optics and Photonics Research Center (CEPOF), Institute of Physics “Gleb Wataghin,” State University of Campinas – UNICAMP, Campinas, São Paulo, Brazil; 2 Microscopy Laboratory Applied to Molecular and Cellular Studies, School of Bioengineering, National University of Entre Ríos, Oro Verde, Entre Ríos, Argentina; 3 Pediatric Endocrinology Laboratory, CIPED, State University of Campinas - UNICAMP, Campinas, São Paulo, Brazil; 4 Department of Medical Genetics, Faculty of Medical Science, State University of Campinas – UNICAMP, Campinas, São Paulo, Brazil; 5 INFABiC - National Institute of Science and Technology on Photonics Applied to Cell Biology, Campinas, São Paulo, Brazil; 6 Department of Structural and Functional Biology, Institute of Biology, State University of Campinas – UNICAMP, Campinas, São Paulo, Brazil; University of Milano-Bicocca, Italy

## Abstract

**Background:**

The confirmatory diagnosis of Osteogenesis Imperfecta (OI) requires invasive, commonly bone biopsy, time consuming and destructive methods. This paper proposes an alternative method using a combination of two-photon excitation fluorescence (TPEF) and second-harmonic generation (SHG) microscopies from easily obtained human skin biopsies. We show that this method can distinguish subtypes of human OI.

**Methodology/Principal Findings:**

Different aspects of collagen microstructure of skin fresh biopsies and standard H&E-stained sections of normal and OI patients (mild and severe forms) were distinguished by TPEF and SHG images. Moreover, important differences between subtypes of OI were identified using different methods of quantification such as collagen density, ratio between collagen and elastic tissue, and gray-level co-occurrence matrix (GLCM) image-pattern analysis. Collagen density was lower in OI dermis, while the SHG/autofluorescence index of the dermis was significantly higher in OI as compared to that of the normal skin. We also showed that the energy value of GLCM texture analysis is useful to discriminate mild from severe OI and from normal skin.

**Conclusions/Significance:**

This work demonstrated that nonlinear microscopy techniques in combination with image-analysis approaches represent a powerful tool to investigate the collagen organization in skin dermis in patients with OI and has the potential to distinguish the different types of OI. The procedure outlined in this paper requires a skin biopsy, which is almost painless as compared to the bone biopsy commonly used in conventional methods. The data presented here complement existing clinical diagnostic techniques and can be used as a diagnostic procedure to confirm the disease, evaluate its severity and treatment efficacy.

## Introduction

Osteogenesis imperfecta (OI), firstly described in the 17th century [1], is a group of inherited connective tissue disorders in which synthesis or structure of type I collagen, is defective and causes bone fragility. This disease has a prevalence of approximately 6–7/100,000 [2]. OI is classified principally into 4 types based on clinical and radiological findings [3], [4]. This classification distinguish type I (mild OI, blue sclerae), type II (perinatally lethal OI), type III (progressively deforming), and type IV (wide range of bone fragility with dentinogenesis imperfecta and normal sclerae).

Although the primary OI clinical presentation is related to defective type I collagen fibers in bone, the defect is also present in other type I collagen-containing tissues such as skin. This means that the disease signature could be readily found in skin, which is more accessible than bone for ex vivo analysis. Skin is also amenable to “topical optical biopsy”. Therefore, in the current study, we present observations of fresh skin biopsies of OI patients. We demonstrated that the combination of two-photon excited fluorescence (TPEF) and second-harmonic generation (SHG) microscopy techniques with different scoring methods can be used not only to discriminate mild or severe OI from normal skin but also to differentiate types and clinical severities of OI.

Historically, clinical and radiological assessments have been the standard to provide diagnosis, classification of disease severity and efficacy of treatment in OI. Confirmation of the disease required bone biopsy, a highly invasive procedure. Nonetheless, the more recent molecular diagnosis of OI are a laborious and time consuming methods, consisting of biochemical measurements and/or histological evaluation of the collagen from biopsy-derived dermal fibroblasts [5]. Therefore, there is a clear need to develop a rapid non invasive method to diagnose OI.

Given that the two main components of dermis (collagen and elastin) can be imaged with SHG and TPEF microscopy, the combination of the two techniques is particularly useful to analyze the structure of the skin in a non invasive way. While TPEF results from the non-linear excitation of molecular fluorescence [6], SHG signals depend on non-linear interactions of illumination with a submicron non-centrosymmetric environment [7], [8]. The non-centrosymmetric structure of fibrillar collagen makes it the major source of the SHG signals in biological materials [9]–[11]. TPEF and SHG microscopy were used to study normal skin [12]–[14] and different pathologies such as cutaneous photoaging [15], [16], psoriasis [13], and selected skin tumors [17]–[20]. Both techniques have already been widely used on ex-vivo tissue samples to perform optical biopsy for morphological characterizations [12], [13], [21]. However, there are only a few reports in the literature using SHG [22], [23] or TPEF+SHG [24] to analyze OI in skin samples.

Campagnola and co-workers have exploited the sensitivity of SHG to identify supramolecular assemblies and to investigate whether this approach could be used to differentiate normal and OI tissues in a murine OI model (mOI) with biochemical and phenotypic characteristics similar to those seen in patients with type III OI. Specifically, they used SHG microscopy (with and without polarization analysis) to show that OIM bone, skin, and tendon were substantially distinct from the wild-type. The SHG intensity as well as morphological differences allowed them to discriminate the cases [22], especially because mOI matrix was comprised of smaller, more randomly packed fibrils, meaning that these structures have a less organized collagen lattice [22], [23]. While the previous studies provided a basis to interpret SHG images of skin and laid the foundation for future *in vivo* optical evaluation of OI, the necessity to improve quantification mechanism capable of distinguishing between different types of OI remained. This requirement is especially important in the first months of life, when the diagnosis based only on the clinical observation of multiple fractures and deformities is somewhat difficult.

The aim of the present work is to show that TPEF+SHG microscopy in human skin provide an OI diagnostic discriminating OI from normal skins and, moreover, that this method is capable to differentiate the clinical types of OI as well. The combination of nonlinear microscopy techniques together with different scoring methods (collagen density, SHG intensity, ratio between collagen and elastic tissue, and texture analysis) can distinguish between the different classical types of OI with great advantages compared to present diagnostic methods, which are primarily clinical with no fast and clear confirmatory method. We believe that the procedure proposed herein is very practical and capable to replace the standard confirmatory method with a new far less invasive method.

## Materials and Methods

### Patients, OI Classification and Protocols

Skin samples from healthy controls and from patients with OI were obtained from the Laboratory of Pediatric Endocrinology, Campinas, SP, Brazil. The research protocol was approved by the local Research Institutional Ethics Committee (Faculty of Medical Sciences, Unicamp, CEP#391/2005) and a written inform consent was obtained from all participants or their parents in accordance with the local Research Ethics Committee. All procedures were in accordance with the Declaration of Helsinki and the ethical principles of the medical community. A total of 9 skin biopsies were analyzed and classified as normal (4 cases) or OI (5 cases). OI patients were classified according to clinical observations in mild OI (Type I - 1 case), moderately affected and severe OI (Type III or Type IV - 4 cases), according with the Sillence classification criteria. Clinical characteristics of these patients and molecular analysis with mutations found in *COL1A1* or *COL1A2* genes confirming the qualitative defects in collagen type I are described in Table 1.

**Table 1:** Patients clinical characteristic and molecular analysis.

Clinical characteristic							Molecular analysis			
Pat.	T.	Age	Severity	Fractures	Bone Def.	Sta.	Gen	E	Mutation	Protein
**A**	I	20	Mild	Infrequent	Absent	N.	*COL1A2*	5	N.A.	N.A.
**B**	IV	42	Moderate	Infrequent	Moderate	Short	*COL1A1*	13	c.859G>A	p.G287S
**C**	IV	12	Moderate	Infrequent	Moderate	Short	*COL1A1*	25	c.1678G>A	p.G560R
**D**	IV	21	Severe	Frequent	Moderate	Short	*COL1A1* *COL1A2*	8 16	c.613C>G c.775G>A	p.P205A p.G259S
**E**	III	21	Severe	Frequent	Severe	Short	*COL1A2*	49	c.3467G>A	p.R1156K

Pat.: Patients, T.: Type, Bone Def.: Bone Deformity, Sta.: Stature, N.: Normal, E.: Exon, N.A.: Not Available.

Normal samples were obtained from eyelid plastic surgery discarded tissue and patient`s biopsy were obtained from growing skin. Fresh skin samples in PBS solution were analyzed by 3D SHG representations within 6 hs of the excision. The other portions of the skin were fixed in 10% formalin, paraffin-embedded, and sequentially cut in 4-µm thick sections. Tissue sections were de-waxed and examined after H&E staining using standard techniques as well as 2D TPEF and SHG.

### Optical Setup and Image Acquisition

**Figure 1:**
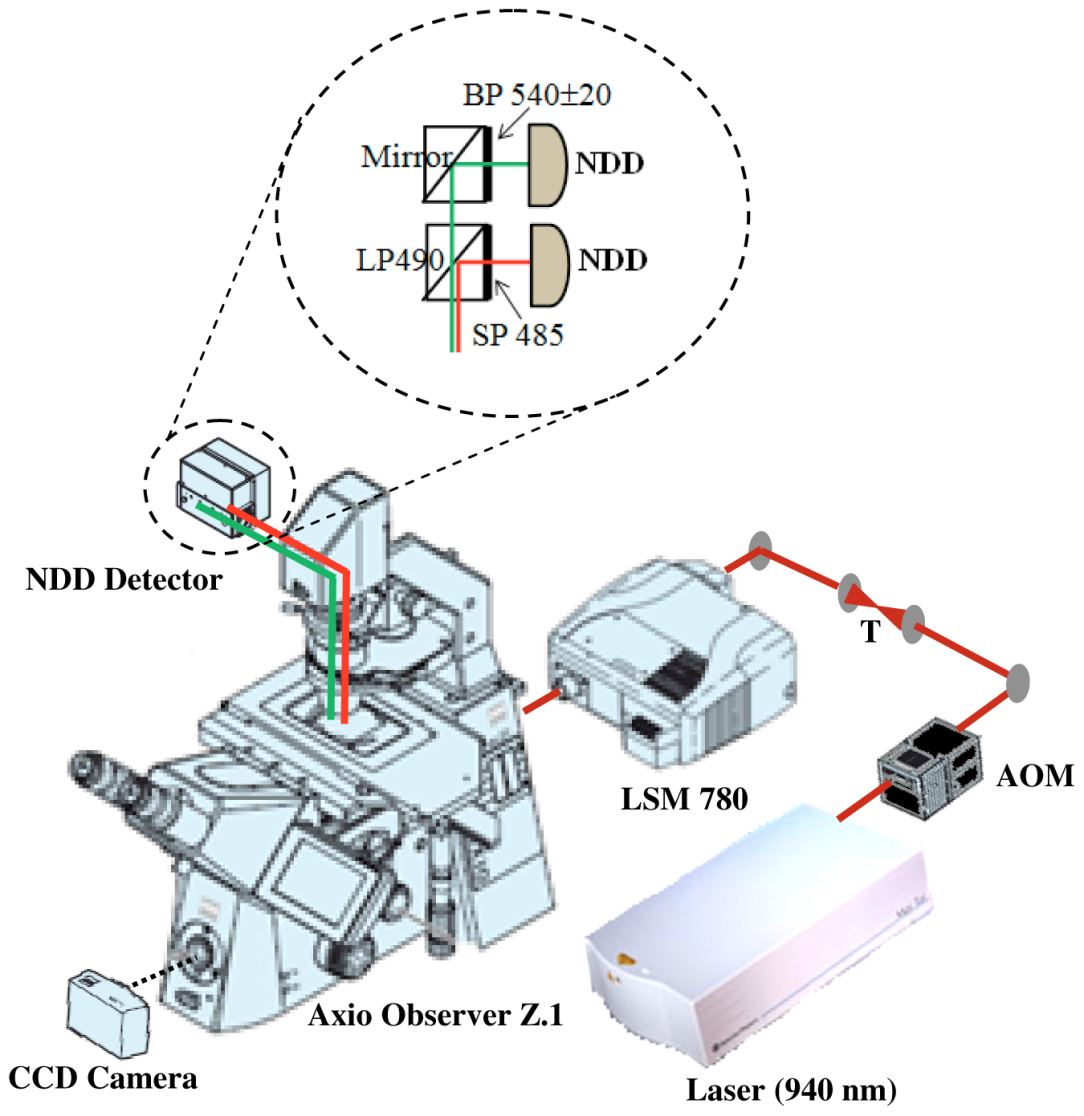
Experimental setup of a nonlinear optical microscopic system. CCD: charge-coupled device, AOM: acoustic-optic modulator, T_1_: telescope, SP: short-pass filter, BP: band-pass filter, LP: long-pass filter, NDD: Non Descanned Detector. The SHG (red lines) and TPEF (green lines) are collected in a transmitted light configuration.

All images were acquired with an inverted microscope Axio Observer Z.1 (Carl Zeiss AG, Germany) equipped with a Zeiss LSM 780-NLO confocal scan head (Carl Zeiss AG, Germany), which was recently described in detail [25], [26]. All accessories were from Carl Zeiss, except when otherwise stated. The excitation beam was provided by a mode-locked Ti:Sapphire laser (Spectra-Physics. Irvine, USA) emitting 100-fs width pulses at a 80-MHz repetition rate. The beam was coupled to the scan head after a collimating telescope. We used circular polarized light to avoid anisotropies for different fibril’s directions. The beam was focused onto the sample by a 40×/1.30 NA oil immersion EC Plan-Neofluar objective. (The forward propagating SHG and two-photon fluorescence signals were collected by the condenser lens 0.55 NA – WD 26 mm and acquired simultaneously by non-descanned detector (NDD) after a blocking filter SP720 (Omega Filters) to prevent back-reflected laser light (Figure 1). In some cases, a mosaic image of the complete tissue section was acquired with a motorized and automatic stage controlled by the Zen2010 software.

TPEF and SHG images were excited with 20 mW (at the sample) of a 940 nm fs beam, generating a SHG at 470 nm and a TPEF >490 nm. A filter cube with a dichroic LP490 nm wavelength and a filter SP485 nm wavelength were placed in front of the SHG detector to cut out unwanted fluorescence light from the SHG channel. Other filter cube with a mirror and a narrow (20 nm FWHM) BP540 filter was used to detect TPEF signal. Wide field H&E images were acquired with a 10 MP digital camera placed on the microscope viewfinder (Figure 1).

### Quantification of Nonlinear Images in Human Skin

Quantitative analysis of the TPEF and SHG images data were performed with ImageJ software (V1.47c NIH, Bethesda USA.).

#### Collagen density

To analyze the depth-dependent decay (DDD) of the SHG signal we took a series of SHG images (354.30 µm×354.30 µm) at 1 µm steps up to a final depth of 40 µm; the average SHG intensity of selected regions at each depth was calculated and plotted as a function of depth. This calculation was performed using Plot Z-axis Profile commands from Image/Stack menu of ImageJ.

Dunn and coworkers demonstrated that the maximum imaging depth is strongly dependent on the absorption and scattering coefficients as well as on the scattering anisotropy [27]. For DDD determination we assume that the transmission of near-infrared light in dermis tissue is absorption dominated. Dunn found that although scattering plays a role in DDD, absorption is the main factor responsible for DDD in turbid media, at least to a depth of focus of 400 µm. Thus, DDD is a good measure of collagen density considering the depths examined in the present study, where other factors, such as direction, distribution, and size of collagen fibers, can be disregarded. The same method has been used previously for the same purpose [28], [29].

#### SHG intensity analysis

We separate SHG signal from noise with a threshold at level 15 from the 0 to 255 gray levels of 8-bit images. Montage of 8 SHG images (1416.64 µm×708.15 µm) were used to obtain a good representation of skin dermis. Then, a square region of interest (ROI) 200×200 µm was drawn and positioned in the image. The coordinates of the square were saved as a ROI file in ImageJ. This positioning was repeated so that 12 non-overlapping ROIs were generated and saved. This array of ROIs was used to assess the entire SHG image on a ROI basis in a consistent manner for each sample in the array. Using the multi-measure plugin, the 12 ROI files were opened and visualized as an overlay of the image, and then the plugin allows separating standard measurements from each of the threshold ROIs. From this data the integrated density (the summation of the pixel intensity values for all of the pixels in the ROI, which is essentially a measure of the total brightness) was quantified.

#### Ratio between collagen and elastic tissue

The second harmonic to autofluorescence aging index of dermis (SAAID) value is a measure of the ratio between collagen and elastic tissue that can be used to evaluate intrinsic and extrinsic skin aging [15], as well as to give a measure of the fibrotic status of the dermis [30]. The SAAID index is defined as:
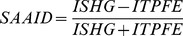
where *I* means the intensity of each signal, SHG/TPEF. If the SAAID index = +1, then collagen fibers are predominant in the dermis; if the index = −1, the elastic fibers predominate over collagen. Intermediate values could be influenced by the characteristic of each tissue. SAAID scoring has resulted in a powerful method to investigate dermis at a large scale (hundreds of µm) [30]; as done in this work, where regions of approximately 1000 µm×400 µm were evaluated.

We obtained the TPEF intensity with the same procedure used for SHG intensity, as above described. SAAID was then calculated only for ROIs mainly comprised of the dermis area. Those ROIs for which 50% or more of area was located in the epidermis were discarded.

#### Texture analysis with gray-level co-occurrence matrix

The gray-level co-occurrence matrix (GLCM) analysis method allowed the classification of different tissues based on the evaluation of geometrical arrangement of collagen. The GLCM is constructed by counting the number of occurrences of a gray level adjacent to another gray level, at a specified pixel distance 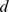 and dividing each counting by the total counting number to obtain a probability. The result is a matrix with rows and columns representing gray levels and elements containing the probability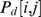 of the gray level co-occurrence between pixels. Usually the matrix is averaged in opposite and different orientations (0–180 deg, 45–225 deg, 90–270 deg, and 135–315 deg) unless a one dimensional feature is dominant and the 0–180 average is sufficient. A detailed explanation on how this matrix in created from the original image can be found in [30]–[32].

From this matrix one can calculate several parameters such as correlation, contrast, energy, and homogeneity. Mathematically the parameters are defined as:
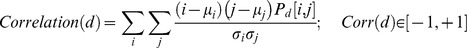


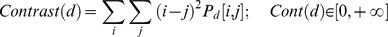


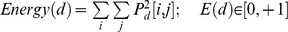


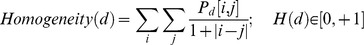
Where 
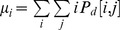
 and 
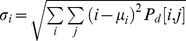
. For 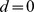 then 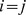 and therefore correlation and homogeneity take the value 1, contrast goes to zero and energy is any number between zero and one. For a homogeneous image where all the entries have the same value *k*, then 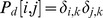 where 
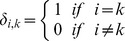
 is the Kronecker delta function. In this case correlation is not defined, contrast is zero, while homogeneity and energy takes the value of 1.

Correlation is a measure of dependence of two different pixel values. If two pixels are well correlated their intensity values are consistent with a well-defined relationship. High correlation occurs in images with periodic features. Contrast indicates the variance of the gray level. High contrast occurs when an image has a high number of pixel pairs with large differences in gray level. Energy is a measure of orderliness of the image (i.e. pixel pair repetitions). Energy is highest in images with uniform gray level or uniform gray-level differences at the specified separation and lower for those with more variation in gray levels. Finally, homogeneity gives information on the similarity of a pixel value in combination with its neighboring pixels to all the other pairs of neighboring pixels in the image. Homogeneity is highest in images with pixels of the same or similar gray levels at the specified separation and orientation.

From the mounted SHG images, we took three images (354.3 µm×354.3 µm) located in the dermis. Non symmetric GLCMs were computed using 256 gray-levels. For a 354.3 µm×354.3 µm (1024×1024 pixels) image, a practical upper bound on pixel separation is 50 pixels. Separations of 1 to 50 in 1-pixel increments were used for this study. Because our collagen fiber orientation changed from sample to sample, we used the four orientations average. The texture analyses were performed with Image-J GLCM-Texture plugin, which was described by Walker and collaborators [33].

### Statistical Analysis

We performed *t*-testing for two-group comparisons. Significance levels employed were non-significant (N.S.), significant (*) *p*<0.05 and very significant (**) *p*<0.01. Data were analyzed with SPSS 10.0 software.

## Results

**Figure 2:**
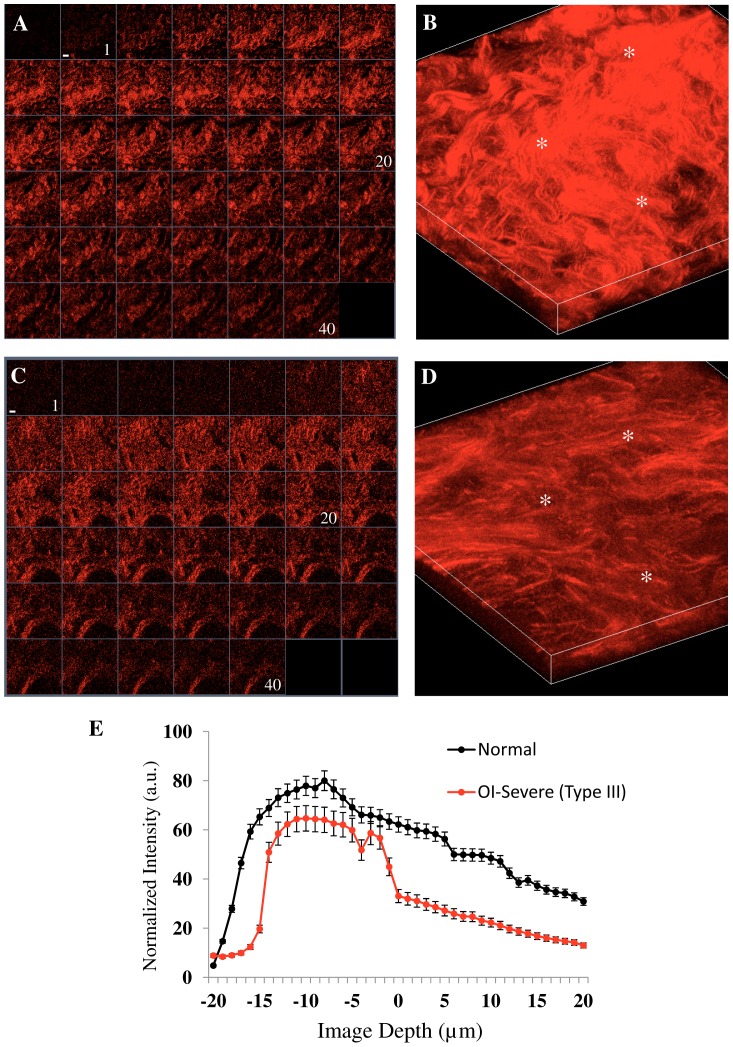
SHG signal in function of skin depth. Representative SHG images from fresh skin biopsies. A, B: normal skin from a control and C,D: Type III OI patient. A and C: gallery view of 40 images at intervals of 1 μm. (1: –20 µm depth, 20: in focus and 40: +20 µm depth). Scale bar = 35 µm. B and D: 3D maximum projection of A and C, respectively. E: depth-dependent decay (DDD) of the SHG signal analyzed to determine the collagen density in normal (black circle) and severe OI-Type III (red circle). The white asterisks indicate the selected regions where the plot was calculated as a function of depth. Each circle represents the mean value and standard deviation of three values corresponding to selected region.

Figure 2 shows representative SHG images of skin from normal and severe OI (Type-III) patients (first and second row, respectively). Fig. 2A and 2C are serial single optical sections and fig. 2B and 2D the corresponding 3D renderings of z series for each tissue. A decrease in SHG signal intensity with increasingly depth can be seen in OI tissues (Fig. 2C) in contrast to the uniform similar collagen intensity in almost all volume examined in normal skin (Fig. 2A). When plotting the depth-dependent decay (DDD) of the SHG signal evaluated at three positions on the stack we found that the OI skin dermis had a significant lower DDD, indicating reduced collagen density (Figure 2E). The highest SHG intensity (>50 a.u.) occurred at about 25 µm in depth (−15 µm to 10 µm) of the normal skin and at 10 µm depth in the OI skin (−10 µm to 0 µm).

**Figure 3:**
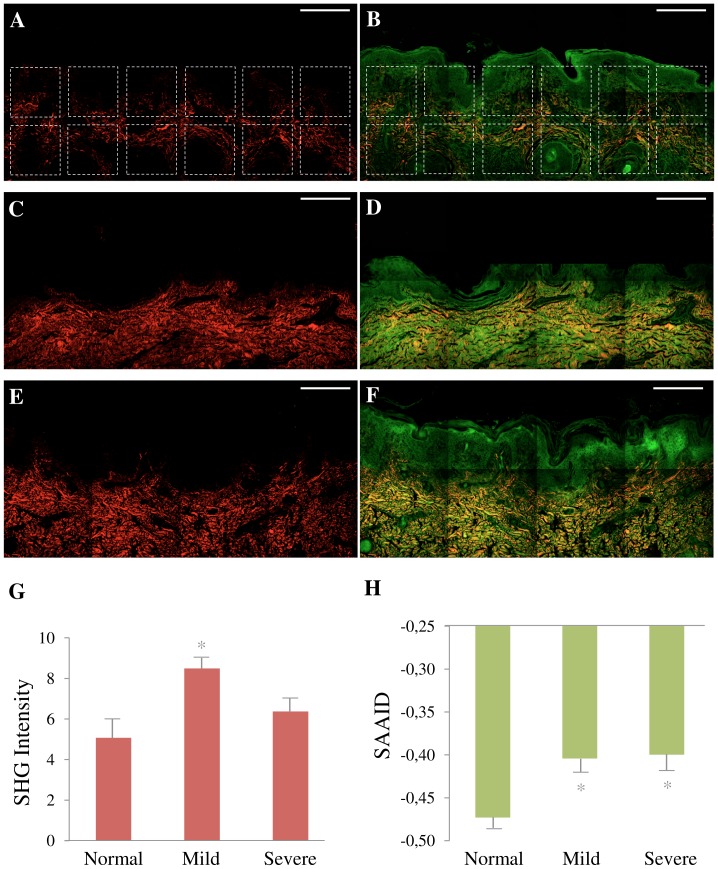
Quantification of collagen and elastic tissues. Montage of SHG (A, C, E) red and TPEF+SHG (B, D, F) green and red images. Representative images of fixed normal skin from control (A, B), Type I OI patient (C, D) and Type III OI patients (E, F). Scale bar = 200 µm. Montage A and B shows the 12 ROI (200×200 µm) selected to perform the quantification of SHG intensity (G) and SHG autofluorescence aging index of dermis (SAAID) (H). These same ROI positioning was used in all images. In G and H, each bar represents the mean ± S.D. of independent measurements. The total number of ROI from which these parameters were extracted was (normal: 85 ROI for 10 images, mild OI: 19 ROI for 3 images, and severe OI: 54 ROI for 8 images). Asterisks indicate a significant (*, p<0.05) difference from normal samples. ROI mainly occupying the dermis area were used.

Next we checked the amount and distribution of collagen in stained histological sections (figure 3). To get a good representative value of collagen content and SAAID index, we analyzed tissue sections using a montage of 8 images using TPEF and SHG images (Fig. 3 A–F) for the determination of collagen content (Fig. 3 G) and SAAID index (Fig. 3 H). Figure 3 shows representative TPEF and SHG images of normal (A, B), mild OI (Type I) (C, D), and severe OI (Type III) (E, F). Comparing these images we can see that collagen (red) content increased and that the intensity of TPEF (green) reduced in OI samples, which is readily noticed by the red/green proportion in the images. To confirm these observations, collagen-related changes were evaluated. These results demonstrated that patients with OI present a slight increase in collagen content (8.49±0.64 mild and 6.37±0.85 severe) as compared to normal skin (5.07±1.03). The differences for mild OI presented statistically significant difference (*p*<0.05, *t*-test) (Fig. 3G). Regarding the elastic tissues, the corresponding SAAID of both OI types presented statistically significant (*p*<0.05, *t*-test) higher values (−0.404±0.040 mild and −0.400±0.050 severe) as compared to normal skin (−0.473±0.06) due to the high SHG (collagen) signal and low TPEF (elastin) signal in this region of dermis (Fig. 3H). In summary, these results show an association between the amount of collagen or elastic tissues and pathological dermis.

**Figure 4:**
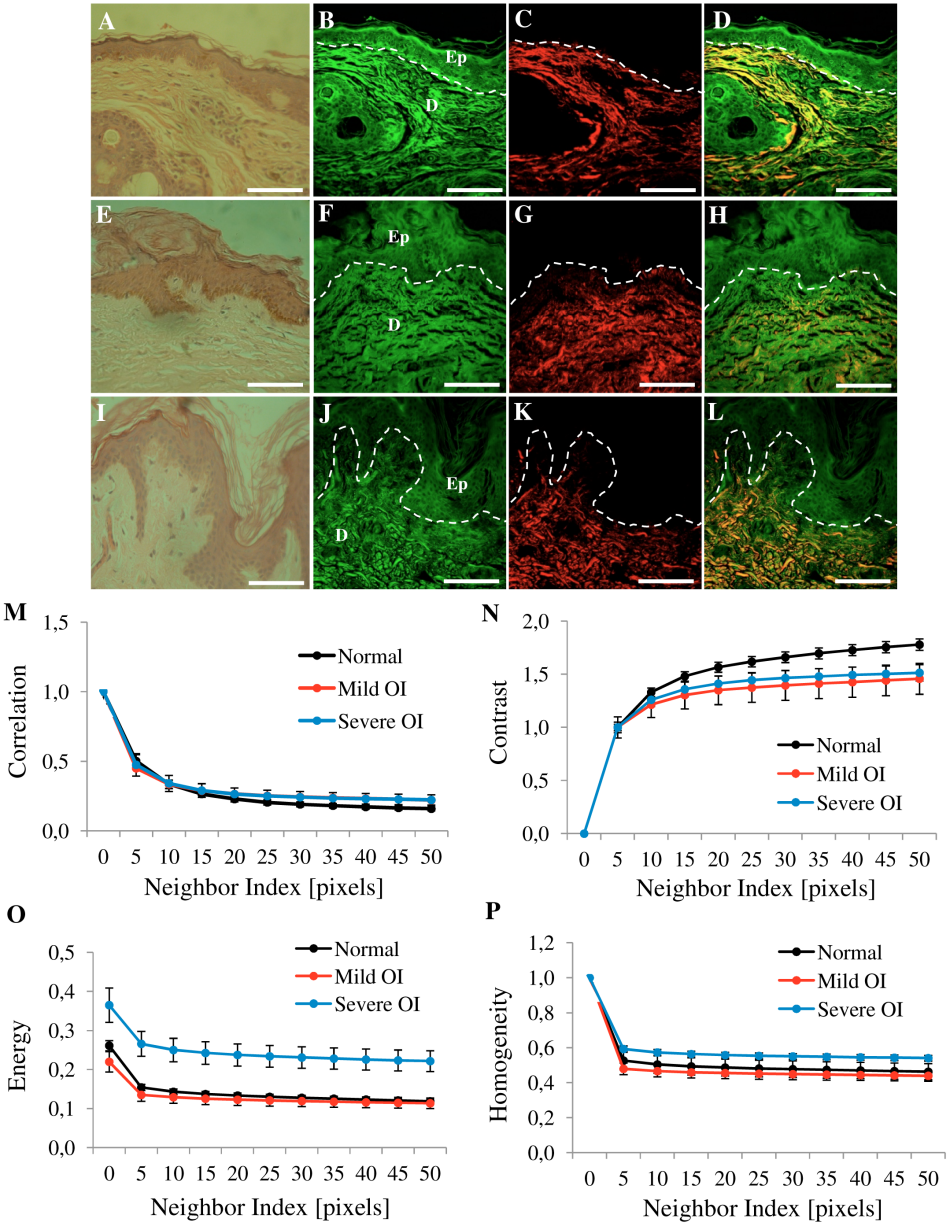
Texture analysis of skin dermis. One representative image (354.30×354.30 µm) of the eight images from figure 3. Normal skin (A, B, C, D), OI-Type I (E, F, G, H) and OI-Type III (I, J, K, L). Figure shows H&E-stained samples (A, E, I), TPEF images in green (B, F, J), SHG images in red (C, G, K) and merge TPEF+SHG images (D, H, L). Epithelial/stromal interface is indicated (white outline). Ep: Epithelium, D: Dermis. Scale bars = 100 µm. Texture analysis (M, N, L, O) using the gray-level co-occurrence matrix (GLCM). Correlation (M), Contrast (N), Energy (L), and Homogeneity (O) values in dermis tissues versus distances pixels; ranging from 1 to 50 pixels (0.35 µm–17.30 µm) in 0, 45, 90 and 135 deg directions of image. (n = 12 normal, n = 3 mild OI, and n = 9 severe OI). Black line (normal), red line (mild OI), and blue line (severe OI).

Besides the difference found in the amount of collagen, a visual inspection of the SHG images of Figure 3A, C, and E reveals that the normal skin have thinner collagen fibers that weave in all directions around the hair follicles. The skin from OI patients presents more variation in collagen fiber thickness when compared to the normal skin. Skin images from the more severe forms of OI (type III and IV) tend to have thicker, broken and wavy collagen fibers that are packed together in the same direction. To quantify these observations we used the texture analyses with gray-level co-occurrence matrix (GLCM). Figure 4 shows representative H&E-stained, TPEF, SHG and merge images from a normal skin (A–D), a type I OI (E–H), and a type III OI patient (I–L) where the correlation (Fig. 4M), contrast (Fig. 4N), energy (Fig. 4L), and homogeneity (Fig. 4O) values were obtained.

More severe OI forms (type III and IV) were best differentiated from normal and from mild OI (type I) by three GLCM parameters: contrast, energy, and homogeneity. Homogeneity and energy gave similar results because they are not completely independent of each other, even if they are related to contrast and orderliness of the image, respectively.

Among the four curves extracted from texture analysis, correlation, contrast, energy, and homogeneity, the energy and contrast at different pixel separations are the most sensitive, with a significant difference (p<0.05) when normal skin was compared with OI patient`s skin. Since collagen fiber width and spacing affects the gray levels transition across the image, it is expected that GLCM energy will change for different collagen morphologies. Normal fibers have lower average energy because they are thinner, causing more variation in gray levels when moving across the image.

**Figure 5:**
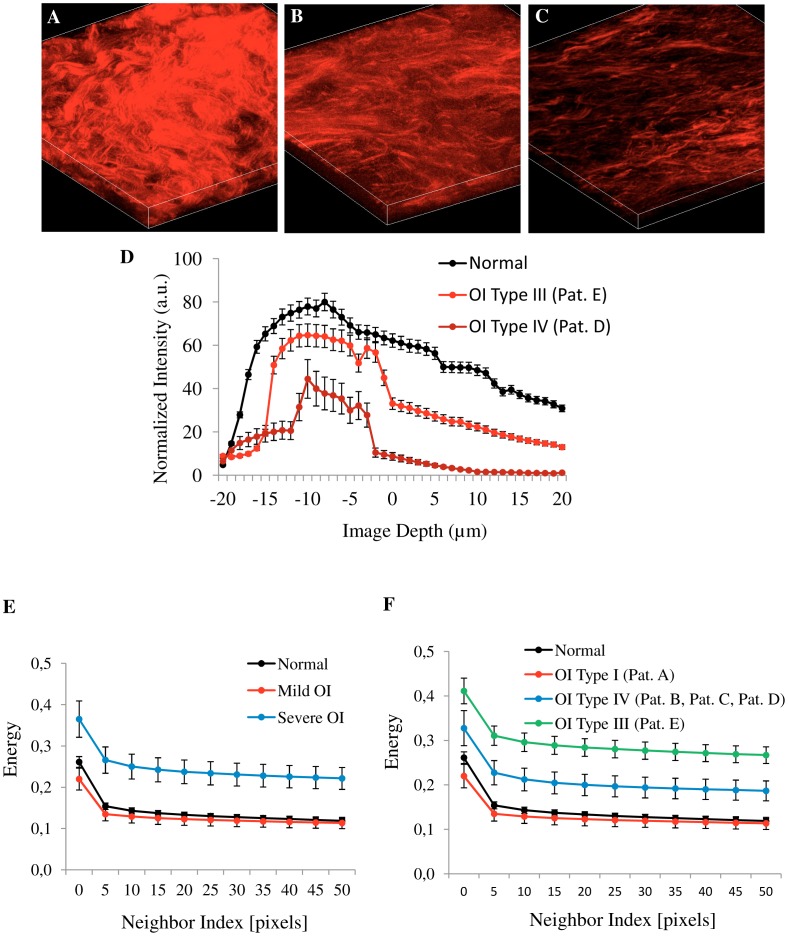
Best methods for quantitative analysis in fresh and fixed skin samples. Representative 3D maximum projection (40 images at intervals of 1 µm) of SHG images from fresh skin biopsies, A: normal skin, B: OI Type III (Patient E) and C: OI Type IV (Patient D). D: depth-dependent decay of the SHG signal analyzed to determine the collagen density in normal (black circle) and severe OI (Type III and IV -red circle). White asterisk indicate the selected regions where the plot was calculated as a function of depth. Each circle represent the mean value and standard deviation of three values corresponding to selected region. Texture analysis (E, F), using the gray-level co-occurrence matrix (GLCM). Energy values was calculated in dermis tissues versus distances pixels; ranging from 1 to 50 pixels (0.35 µm - 17.30 µm) in 0, 45, 90 and 135 deg directions of image (E: n = 12 normal, n = 3 mild OI, and n = 12 severe OI; and F: n = 12 normal, n = 3 OI Type I, n = 9 OI Type IV, n = 3 OI Type III). Pat: patients.

The most important fact was that the two scoring methods (collagen density and energy-GLCM) (Figure 5) were capable to discriminate satisfactorily the different OI patients according to their clinical severity. Using fresh biopsies one could detect a marked decrease in the density of the network of collagen fibers in the 3D representations of SHG images from severe OI patient’s samples (Fig. 5B (Type III) and Fig. 5C (Type IV)), when compared with the 3D representation of SHG images from normal skin fresh biopsies (Fig. 5A). This was confirmed quantitatively by the DDD of the SHG signal evaluated at three positions on the stack (Fig. 5D), confirming that severe OI is less densely packed. Furthermore, energy value of GLCM texture analysis not only separated type I and type III OI samples from normal skin (Fig. 5E) but it can also differentiate (with statistical significance) between patients with varying degrees of OI, including type IV OI (Fig. 5F).

**Figure 6:**
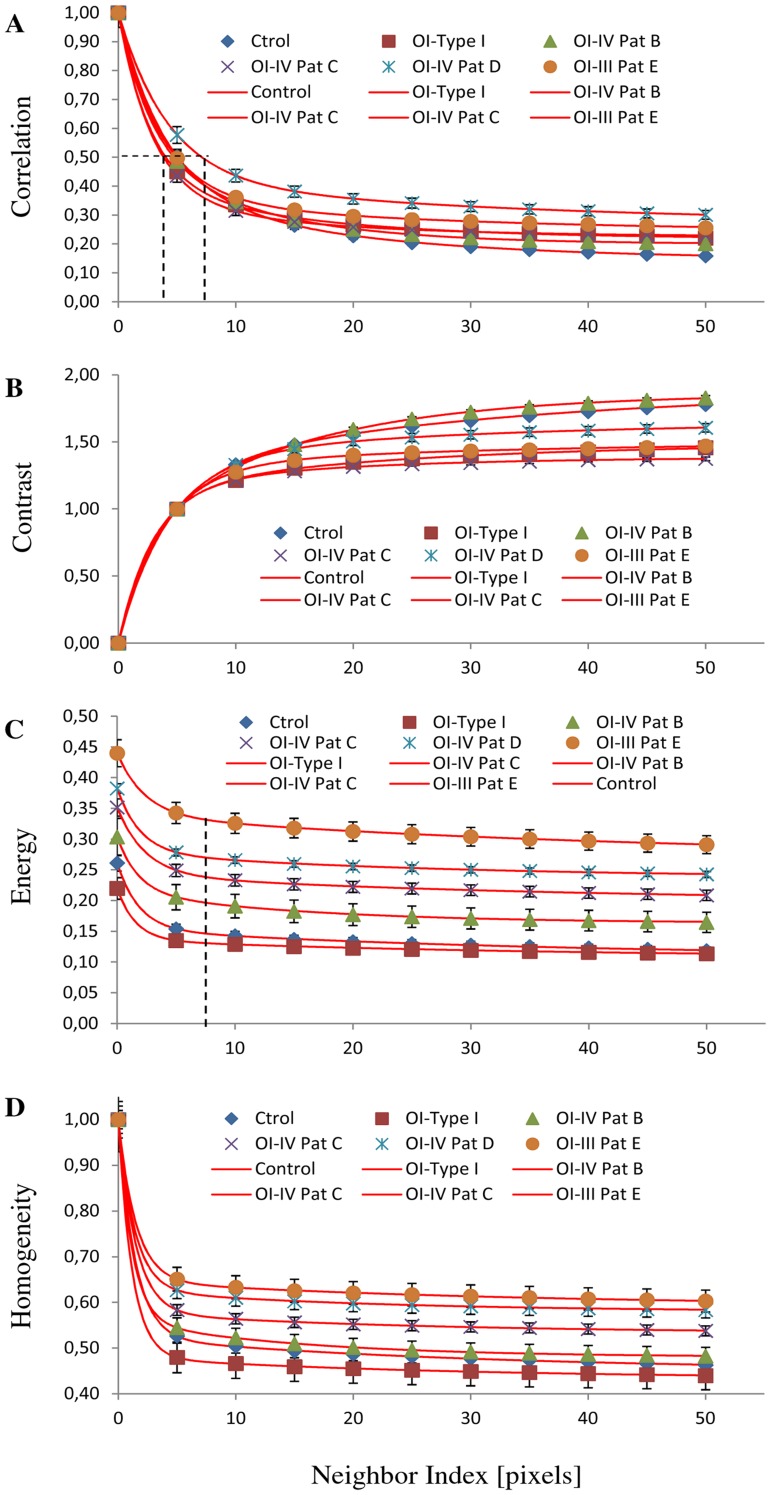
Texture analysis in all patients. Texture analysis using the gray-level co-occurrence matrix (GLCM). The plots were fit (red curve) with a constant background of two exponentials using the following formula y = a_0_+ a_1_^.^ e ^(−1×/×1)^+a_2_^.^ e^(−1×/×2)^. Correlation (A), Contrast (B), Energy (C), and Homogeneity (D) values in dermis tissues versus distances pixels; ranging from 1 to 50 pixels (0.35 µm − 17.30 µm) in 0, 45, 90 and 135 deg directions of image. (n = 12 normal, n = 3 OI Type I Patient A, n = 3 OI Type IV Patient B, n = 3 OI Type IV Patient C, n = 3 OI Type IV Patient D, and n = 3 OI Type III Patient E). Pat: patients.

In more precise quantitative terms, we know that collagen fibers in dermis have a diameter in the range between 0.5 to 3 µm. Therefore we should expect that GLCM analysis would show a repeating structure with a distance of about 1.5–8 pixels correspondingly to the 0.5–3 µm range. We showed that OI patients presented thicker fibers, therefore GLCM correlation signal is expected to drop on a longer scale. The values of decay length were obtained by fitting the correlation data with a double exponential decay function (red curve, Fig. 6). The decay length values using the Corr_50_ (the pixel distance where the correlation dropped below 50% of the initial value) obtained are among 1.3 to 2.5 µm (3.8 - 7 pixels) (Fig. 6A) confirming that patient D had thicker collagen fiber ≈ 2.5 µm (7 pixels). Using this pixel distance as comparison, the Energy parameter show the following values 0.15±0.02 (Normal), 0.13±0.04 (Pat. A), 0.20±0.03 (Pat. B), 0.24±0.04 (Pat. C), 0.29±0.03 (Pat. D), and 0.33±0.04 (Pat. E), with a significant difference (p<0.05) when normal skin was compared with OI patient`s (B,C,D and E) skin. This means that this texture parameter allowed the identification of each patient pattern clearly (Fig. 6C). Interestingly, one of type IV patient presented a more severe phenotype (Patient D) than the others, with a degree of bone fragility that limits her mobility and confines her to a wheelchair. SHG image of this patient´s skin showed a more compromised collagen pattern (thicker collagen fiber), intermediate between type III and the two other type IV patients. Indeed, molecular analysis of this patient showed that she had two heterozygous mutations, one in *COL1A1* and another in *COL1A2* gene.

Taken together the results allow the conclusion that these nonlinear microscopy techniques in association with specific scoring methods (collagen density and energy-GLCM) are excellent diagnostic tools to distinguish the different types of OI in human skin and have a good correlation with patient`s phenotype severity.

## Discussion

Our results on the OI human skin show that the combination of measurements of the collagen density and energy-GLCM pattern provides a quantitative method to differentiate healthy and diseased tissues. First, we demonstrated that collagen density metrics was useful to differentiate severe OI from normal skin. Reduced collagen content with depth in the disease tissue was recorded. This result is similar to quantifications previously performed on skin of murine model of OI [22]. Moreover this is an easy and fast method which can be applied to thick and fresh tissue. 3D images can be obtained without fixation, sectioning or staining. Furthermore, as demonstrated in Fig. 2 and Fig. 5A–D, SHG provides higher-resolution and higher-contrast visualizations of the collagen assembly than the ordinary observation after histological staining. It is difficult to observe the variation of collagen content with depth in tissue sections for which a 3D reconstruction based on serial sections is much more complicate then a 3D reconstruction with deep nonlinear confocal microscopy.

In this study we found no significant differences in the intensity of collagen (Fig. 3G) between severe OI and normal skin. However we found a reduction of elastic component in the dermis of both, mild and severe OI, compared with normal tissues. This explains the more positive value of SAAID index (Fig. 3H). Correlation plot shows that decay curve was similar in all samples analyzed (Fig. 4M), revealing that collagen fibers are organized similarly and with certain periodicity. However, we were able to demonstrate using texture analysis that, even if both mild and severe OI have fibrillar collagen similarly organized in fibers and bundles, the dimension of individual fibers differs between OI types.

Moreover, we observed statistically significant differences in contrast (Fig. 4N) and energy values (Fig. 4O). Normal skin image show higher contrast values because it has a high number of pixel pairs with large differences in gray level occurring at the specified separation and orientation while the OI skin image present more pixels with similar gray level. This similarity in gray level was confirmed with energy and homogeneity values (Fig. 4O, P). Severe OI presented higher energy (low variation in gray levels) with respect to normal and mild OI (high variation in gray levels). Therefore, this approach allows us to use quantitative comparisons of the structural organization based on differences of the collagen fiber width and spacing. More compact collagen fibers results in higher energy values. Using energy values, it was possible to distinguish, not only OI disease from normal skin, but also to discriminate the different types of OI (Fig. 5F and Fig. 6C). To date, OI diagnosis is based on clinical follow up of patients radiological and densitometric evaluations. There is clinical variability within the same OI type. The diagnosis classification does not take these differences into consideration. In this way, despite the small number of patients, we were able to demonstrate that these optical techniques can be used not only to discriminate mild or severe OI from normal skin but also to prove a principle of a diagnostic method to differentiate from an collagen signature (with statistical significance) the different types and clinical severities of OI.

In summary, we showed that SHG could differentiate between normal and OI human tissues based on the collagen density in the whole fresh tissue biopsies. Energy-GLCM value demonstrated that we can distinguish between OI type III and Type IV. The non linear optics approach has significant advantages over conventional imaging methods because it enables the optical sectioning in deep tissues and, also, because it allows the acquisition of both TPEF to visualize skin elastic components and SHG to reveal the collagen supramolecular structure. These are label free techniques without interferences of exogenous markers. This approach may be particularly valuable in diagnosing the severity of OI and correlating this with genetic information. We propose that this method could complement the existing clinical diagnosis (see Table 2) and be used as a diagnostic tool or as a measurement of treatment efficacy.

**Table 2:** Postnatal and SHG diagnosis of OI.

Types	Clinical Diagnosis					SHG Diagnosis		
	Severity	C. Fractures	Stature	Bone D.	DI	DDD	SAAID	GLCMEnergy Value (*d* = 7)
**N**						High	Negative	Low (0.15)
**I**	Mild	No	Normal	Rarely	Variable	N.A.	Negative	Low (0.13)
**II**	Severe Lethal	Yes	Severely Short	Very Severe	N.A.	N.A.	N.A.	N.A.
**III**	Severe	Yes	Very Short	Moderate Severe	Yes	Low	More Positive	High (0.33)
**IV**	Moderate	Rarely	Variable Short	Mild Moderate	Variable	Low	More Positive	Medium (0.24)

N.: Normal, C. Fractures: Congenital Fractures, Bone D.: Bone Deformity, N.A.: Not Available, DI: Dentinogenesis Imperfecta, DDD: Depth-dependent decay, SAAID: SHG autofluorescence aging index of dermis, GLCM: Gray-level co-occurrence matrix, *d*: pixel distance.
